# Self-supervised monocular depth estimation for colonoscopy using normal-guided cross-attention

**DOI:** 10.1007/s11548-026-03720-1

**Published:** 2026-08-13

**Authors:** Yahui Zhang, Stamatia Giannarou, Daniel S. Elson

**Affiliations:** The Hamlyn Centre for Robotic Surgery, Department of Surgery and Cancer, Imperial College London, London, SW7 2AZ UK

**Keywords:** Monocular depth, Self-supervision, Normal, Cross-attention

## Abstract

**Purpose:**

Reliable 3D reconstruction of colonoscopic scenes can facilitate navigation and enhance lesion assessment by indicating the regions that have been inspected and revealing the geometric structure of polyps. However, accurate depth estimation in colonoscopy remains highly challenging due to specular highlights and low-texture areas. This study aims to improve monocular depth estimation in colonoscopy by incorporating surface normal information.

**Methods:**

We propose a self-supervised deep learning method for relative depth estimation that integrates surface normal information through a cross-attention mechanism. Surface normals are first predicted by an existing normal estimation model and then used as auxiliary geometric priors. The depth estimation network employs cross-attention to adaptively fuse normal features with image features, enabling spatially selective geometric refinement.

**Results:**

Quantitative and qualitative comparisons show that the proposed method outperforms state-of-the-art self-supervised methods on SimCol3D, C3VD, and real colonoscopy data. The proposed method generates more realistic depth maps, preserving mucosal folds and lumen geometry. Ablation studies verify that the cross-attention module is crucial for effectively exploiting normal information for accurate depth estimation.

**Conclusion:**

By leveraging cross-attention to fuse predicted surface normals with image features, the proposed method enhances monocular depth estimation accuracy and robustness in colonoscopy. This approach provides a general and lightweight strategy for incorporating geometric priors into self-supervised frameworks, offering potential benefits for downstream tasks such as 3D reconstruction and endoscopic navigation.

## Introduction

Colorectal cancer is among the most common and serious malignancies worldwide. Colonoscopy remains the clinical gold standard for detection, but its effectiveness depends on the visual judgment and manual operation of the endoscopist. The complex structure of the colon and challenging lighting conditions often make visual inspection difficult, resulting in missed polyps. Deep learning has the potential to alleviate these limitations by enhancing spatial understanding through three-dimensional (3D) reconstruction. Accurate depth estimation during colonoscopy can improve navigation, assist in identifying unexamined areas, and support the assessment of polyps.

In recent years, monocular depth estimation has achieved remarkable progress with the rapid advancement of deep learning. A wide range of self-supervised approaches have demonstrated that reliable depth maps can be inferred from monocular images without requiring ground-truth annotations, by exploiting photometric consistency across consecutive frames. These techniques have proven effective in natural outdoor or indoor scenes, where texture variation, rigid motion, and stable illumination provide sufficient geometric cues.

However, such assumptions do not hold in colonoscopy. In endoscopic imaging, specular highlights, illumination changes, and low-texture mucosal surfaces introduce ambiguities between reflectance and geometry. Furthermore, the non-Lambertian appearance of tissue causes photometric losses to correlate poorly with actual depth. As a result, existing monocular depth estimation methods trained for natural scenes may fail to recover accurate geometry in endoscopic environments, producing unstable predictions.

In this paper, we present a novel self-supervised framework for monocular depth estimation in colonoscopy that incorporates surface normal information as geometric guidance. Instead of relying solely on photometric consistency or enforcing explicit consistency constraints, we leverage surface normals as auxiliary priors to guide the learning of structural depth features. This design reduces the influence of illumination changes and improves the geometric reliability of the predicted depth. To effectively integrate normal information, we propose a cross-attention mechanism that adaptively fuses the spatial and semantic relationships between surface normals and image features. This enables the network to selectively focus on geometry-relevant regions, mitigating the effect of specularities or low texture. In summary, our contributions are as follows:

1. We propose a novel self-supervised framework for monocular depth estimation in colonoscopy that incorporates surface normal for auxiliary geometric guidance.
2. We design a cross-attention fusion module to adaptively integrate surface normal and image features, enabling the model to focus on geometrically informative regions.
3. We perform experiments on the Simcol3D and C3VD datasets, and on real colonoscopy images that demonstrate that our method achieves superior accuracy and visual quality compared with current self-supervised approaches, and effectively mitigates the impact of specular highlights.

**Fig. 1:**
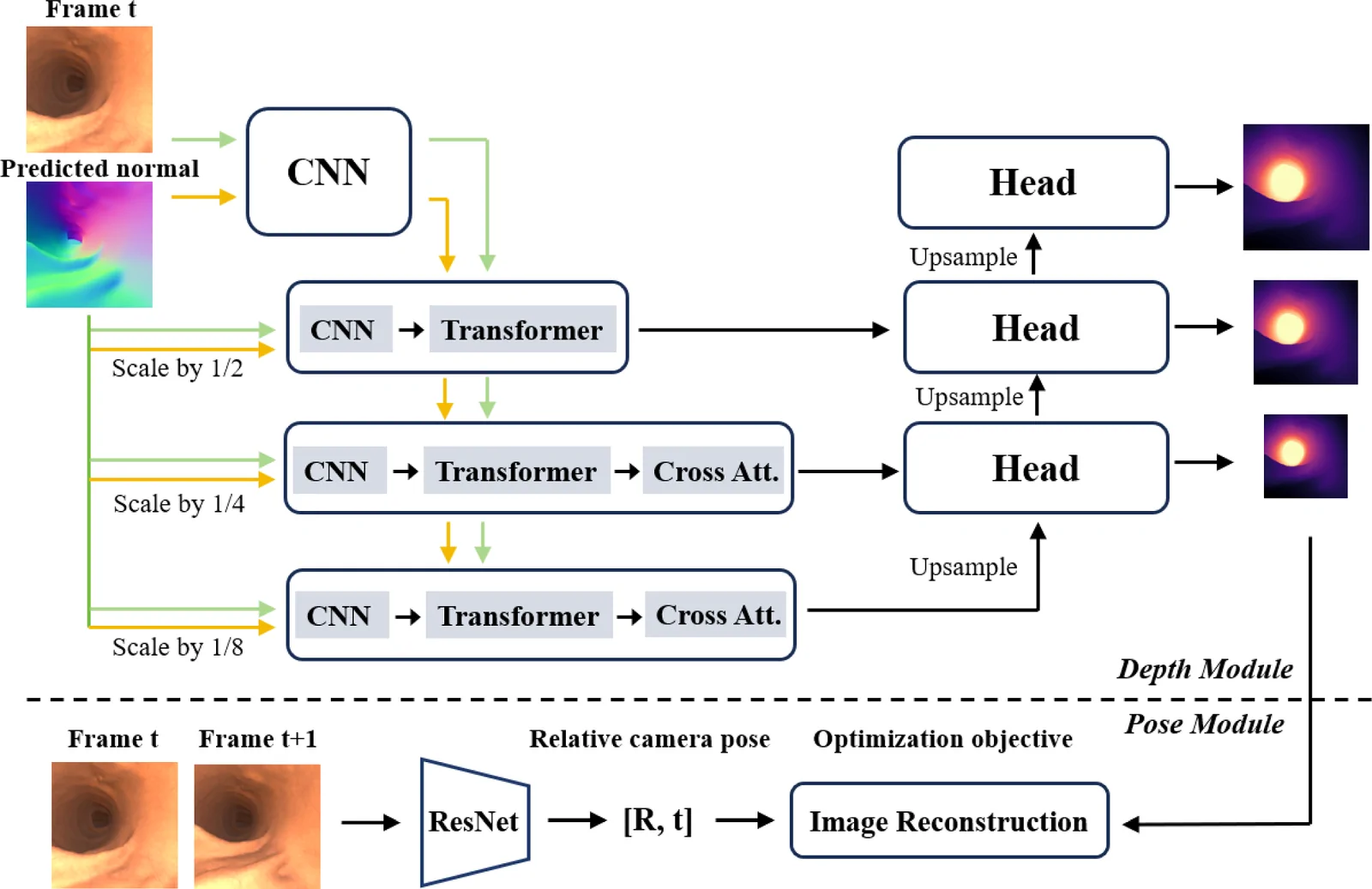
Overview of the proposed architecture. The model consists of two key components: a depth module that predicts depth maps at multiple scales, and a pose module that estimates the relative camera pose between consecutive input frames. The predicted pose is then used to warp the neighboring frames for image reconstruction, which serves as the self-supervised training objective

## Related work

Monocular depth estimation in natural scenes has achieved remarkable progress in recent years. Supervised learning methods have demonstrated high accuracy when large-scale datasets with ground-truth depth are available [1–3]. However, acquiring dense and reliable depth annotations in real-world environments is expensive and time-consuming. As a result, much research has focused on self-supervised approaches. Zhou et al. [4] proposed an unsupervised method for monocular depth estimation from video sequences. They combined a depth and a pose network, trained via a view-reconstruction loss between adjacent frames. Motivated by this idea, other works have extended and refined this framework to achieve higher accuracy in self-supervised monocular depth estimation. Godard et al. [5] presented a multi-scale sampling strategy to reduce artifacts and an auto-masking scheme to exclude invalid pixels. Building on the success of Vision Transformers, Zhao et al. [6] designed a self-supervised depth estimation framework that fused local convolutional features with global transformer representations, achieving superior performance. To reduce the computational cost caused by the Transformer, Zhang et al. [7] proposed a hybrid CNN–Transformer framework that combined the dilated convolutions and features interaction modules for efficient local–global feature fusion.

However, these methods for natural scenes may not be directly applicable to colonoscopy scenes, as the imaging conditions differ. Colonoscopy images often exhibit strong specular reflections, regions of low texture, and complex illumination patterns, making accurate monocular depth estimation more challenging.

In response to these challenges, Liu et al. [8] utilized sparse depth cues from Structure from Motion for weak supervision along with flow and depth consistency losses for optimization. Ozyoruk et al. [9] introduced an affine-based brightness adjustment to improve robustness under illumination changes for depth estimation. To alleviate the effects of specular reflections, Daher et al. [10] proposed a self-supervised framework that decomposed images into shading, albedo, depth, and specular components using a non-Lambertian model to disentangle reflections and improve reconstruction accuracy. Liu et al. [11] developed a U-shaped network that incorporates spatial and channel attention mechanisms, enabling brightness- and color-invariant learning and improved depth accuracy through multi-scale feature extraction. Shao et al. [12] proposed a self-supervised depth and motion estimation approach that modeled variations in illumination via appearance-aware flow and relaxed traditional brightness constancy assumptions, dealing with brightness fluctuations in endoscopic videos. Cui et al. [13] adapted large pre-trained models for self-supervised depth estimation in endoscopic scenes by fine-tuning a minimal set of parameters, enhancing sensitivity to high-frequency visual details.

Surface normal information provides valuable geometric cues that can enhance the accuracy and smoothness of depth estimation. In natural scenes, the NASDE method [14] incorporates normal estimation as an intermediate supervision signal, enforcing consistency between the predicted depth and surface normal to achieve 3D reconstruction. Similarly, Zhan et al. [15] utilized consecutive stereo image pairs to jointly predict depth and surface normals for a reference image, and regularize the depth estimation through a geometric consistency-based loss. In endoscopic scenarios, Esteban et al. [16] also explicitly enforced normal-depth consistency to refine the depth estimation. Li et al. [17] proposed to refine depth in highlighted regions by constraining surface normals through reflection geometry and further aligned illumination between frames using an endoscopic spotlight model to maintain consistent appearance for self-supervised learning. However, this approach explicitly relied on normal computation, which may introduce errors under strong reflections or incomplete depth cues. To address this limitation, we adopt an implicit strategy that integrates normal features into image representations via cross-attention, allowing the network to capture geometric consistency.

## Methodology

The architecture of the proposed framework is illustrated in Fig. 1, consisting of a depth module and a pose module. In the depth module, surface normal maps are first estimated using an off-the-shelf model [18] and used as geometric priors to guide depth estimation. Unlike current methods that jointly estimate depth and normals or enforce explicit depth-normal consistency, the predicted surface normals are treated as auxiliary geometric cues to regularize relative depth estimation. Image and surface normal features are then projected into a shared embedding space and fused through a cross-attention block, enabling the model to effectively align appearance cues with geometric structure. The pose module estimates the relative camera poses between consecutive frames, which is then used to warp neighboring views for image reconstruction. Finally, the reconstruction loss between the synthesized and target frames serves as the self-supervised objective, allowing relative depth and camera pose to be jointly optimized without ground-truth supervision.

### Depth module

**Fig. 2:**
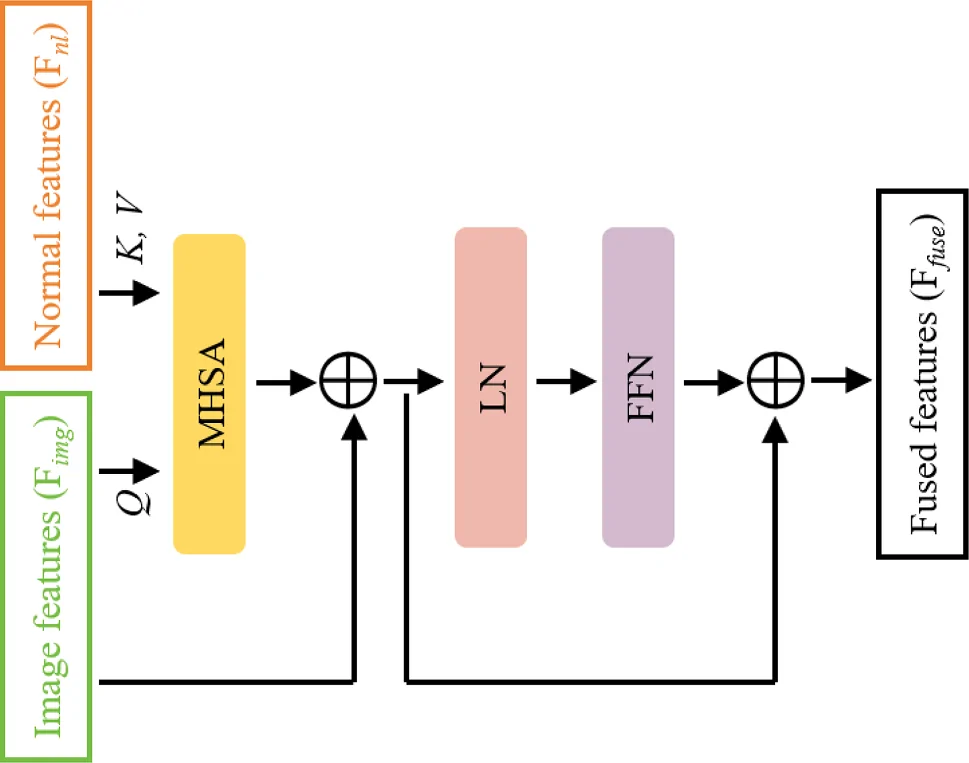
Normal-based cross-attention mechanism. The multi-head cross-attention (MHSA) module uses image features as query (*Q*) and normal features as key (*K*) and value (*V*), integrating geometric cues into image representations. The fused features are refined via residual connections, layer normalization (LN), and a feed-forward network (FFN)

*Encoder.* The first stage of our framework focuses on extracting hierarchical representations from input images and surface normal maps. Following the lightweight encoder design proposed in [19], a hybrid structure was designed that integrates convolutional operations and transformer-based attention to balance efficiency and contextual representation. Specifically, each encoder stage contains several consecutive dilated convolutional layers with varying dilation rates, which expand the receptive field and capture multi-scale contextual features. Transformer-based attention blocks are then integrated after the convolutional layers to model long-range relations. To reduce the computational cost, a simplified attention block is adopted that linearly projects the feature map into queries, keys, and values within the same embedding space. The feature interactions are modeled through a cross-attention mechanism, which adaptively reweights spatial responses according to the global correlations while preserving channel efficiency. The resulting features are refined through residual connections, layer normalization, and a lightweight feed-forward sublayer to enhance nonlinear expressiveness. The image and predicted surface normals are processed by separate encoders to extract modality-specific features (Fig. 2).

*Feature fusion.* We introduce a normal-based cross-attention mechanism to fuse geometric cues from estimated surface normals into the image representation. Specifically, the image encoder produces feature maps $\textbf{F}_{img} \in \mathbb {R}^{B \times C \times H \times W}$, while the normal estimation branch outputs surface normal features $\textbf{F}_{nl} \in \mathbb {R}^{B \times C \times H \times W}$. Both feature maps are flattened along the spatial dimension to obtain the query $(\textbf{F}_{img})$, key $(\textbf{F}_{nl})$, and value $(\textbf{F}_{nl})$ representations.

The multi-head attention cross-operation is then performed as:

1 $$\begin{aligned} \text {Attention}(Q, K, V) = \text {Softmax}\left( \frac{QK^\top }{\sqrt{d_k}}\right) V, \end{aligned}$$

where $d_k$ denotes the feature dimension of each attention head. This process enables the image features to query the geometric context encoded by the normal features, allowing the network to selectively emphasize structure-consistent information in reflective or textureless regions. The attention output is fused with the original image features through a residual connection, followed by a feed-forward network (FFN) for feature refinement:

2 $$\begin{aligned} \textbf{F}_{out} = \textbf{F}_{img} + \text {FFN}\big ( \text {LN}(\textbf{F}_{img} + \text {Attention}(Q,K,V)) \big ).\quad \end{aligned}$$

By incorporating normal-guided cross-attention, the proposed framework learns geometry-consistent depth features that are more robust to specularities and illumination variations.

*Decoder.* The decoder adopts a UNet–shaped architecture that progressively reconstructs depth maps from the fused image-normal features. It employs convolutional layers with skip connections to effectively integrate multi-scale features from different encoder stages. Feature upsampling is subsequently performed to recover spatial resolution and preserve fine structural details. To enable multi-scale depth estimation, three prediction heads are attached to the decoder, each consisting of a convolutional layer, bilinear upsampling, and a sigmoid activation to produce inverse depth maps at different resolutions. All predicted depth maps are jointly optimized in the self-supervised training process to ensure consistent multi-scale depth estimation.

### Pose module

The pose module aims to estimate the relative camera motion between consecutive frames, which is essential for reconstructing the scene geometry and enforcing view consistency constraints. This module takes two consecutive frames $\textbf{I}_t$ and $\textbf{I}_{t+1}$ as inputs and predicts the corresponding camera pose comprising rotation $\textbf{R}_{t \rightarrow t+1}$ and translation $\textbf{t}_{t \rightarrow t+1}$.

To achieve this, the two frames are concatenated along the channel dimension and fed into a convolutional backbone based on a ResNet variant.

3 $$\begin{aligned} {[}\textbf{R}_{t \rightarrow t+1},\, \textbf{t}_{t \rightarrow t+1}] = \text {ResNet}([\textbf{I}_t, \textbf{I}_{t+1}]). \end{aligned}$$

To balance prediction accuracy and computational efficiency, we employ ResNet-18 as the backbone of the pose module.

### Training

To enable training without using ground-truth depth, we adopt a self-supervised strategy based on photometric consistency between adjacent frames. Given the predicted depth map $D_t$ of the current frame $\textbf{I}_t$ and the estimated relative camera pose $(\textbf{R}_{t \rightarrow t+1}, \textbf{t}_{t \rightarrow t+1})$, the neighboring frame $\textbf{I}_{t+1}$ can be geometrically reconstructed by warping $\textbf{I}_t$ into the target viewpoint. The warping operation is implemented through differentiable image sampling, which projects each pixel in $\textbf{I}_t$ into the coordinate system of $\textbf{I}_{t+1}$ using the predicted depth, camera pose, and intrinsics. The reconstructed image $\hat{\textbf{I}}_{t+1}$ is then compared with the actual frame $\textbf{I}_{t+1}$, and the photometric difference $\mathcal {L}_{photo}$ serves as the supervision signal for depth and camera pose learning.

4 $$\begin{aligned} \mathcal {L}_{photo}= &   \frac{1}{N} \sum _{p} \Big [ \alpha \, \frac{1 - \text {SSIM}(\textbf{I}_{t+1}(p), \hat{\textbf{I}}_{t+1}(p))}{2} \nonumber \\  &   \quad + (1 - \alpha ) \, \Vert \textbf{I}_{t+1}(p) - \hat{\textbf{I}}_{t+1}(p) \Vert _1 \Big ]. \end{aligned}$$

To encourage spatial consistency in the predicted depth maps and suppress noisy fluctuations in textureless areas, an edge-aware smoothness term $\mathcal {L}_{smooth}$ is introduced.

5 $$\begin{aligned} \mathcal {L}_{smooth} = \frac{1}{N} \sum _{p} \Big ( |\partial _x D_t(p)| \, e^{-|\partial _x \textbf{I}_t(p)|} + |\partial _y D_t(p)| \, e^{-|\partial _y \textbf{I}_t(p)|} \Big ).\quad \end{aligned}$$

Following previous work [17], the specular highlights in $\textbf{I}_t$ are removed in this process. Therefore, the total loss is defined as follows:

6 $$\begin{aligned} \mathcal {L}_{total} = \mathcal {L}_{photo} + \lambda _{s} \mathcal {L}_{smooth}. \end{aligned}$$

## Experiments

### Dataset

*SimCol3D* [20]. This dataset was developed for depth and camera pose estimation in colonoscopy. It contains 37.8K RGB images with a resolution of 475×475. Following [20], we divide the data into training and testing subsets at the sequence level. Specifically, the SyntheticColon_I dataset is used in our experiments, with 11 sequences allocated for training, one sequence (Frames_S14) for validation, and the remaining three sequences reserved for testing.

*C3VD* [21]. This is a colonoscopy dataset comprising 10,015 frames generated from 22 video sequences. Each frame provides ground-truth depth, surface normal, optical flow, camera pose, and corresponding 3D models. The dataset was rendered using a wide field-of-view (FOV) camera, resulting in image distortion that closely resembles real endoscopic conditions. We adopt the training/testing split used in [10]. Specifically, we used 18 sequences for training, 3 sequences for testing, and 1 sequence (cecum_t3_a) for validation to ensure that no overlap occurred between the two sets.

### Evaluation metrics

Following existing work in monocular depth estimation for endoscopy [13, 17], we adopted a standard evaluation protocol to quantitatively assess the performance of the proposed method. The evaluation metrics included the commonly used AbsRel, Sq Rel, RMSE, and RMSE log, along with threshold-based accuracy using δ<1.25, $\delta < 1.25^2$, and $\delta < 1.25^3$.

We evaluated the proposed method under two complementary settings: (i) in-domain performance and (ii) cross-dataset generalization via cross-dataset transfer. Specifically, we report results when training on SimCol3D and testing on C3VD for cross-dataset generalization.

### Implementation details

All experiments were conducted on NVIDIA RTX 5000 GPUs with a batch size of 12. The AdamW optimizer was adopted with a weight decay of 0.01 and an initial learning rate of $5\times 10^{-4}$. The learning rate followed a cosine annealing schedule, and the total number of training epochs was set to 30. The loss coefficients, α and $\lambda _{s}$, were defined as 0.85 and 0.001. The input normal was obtained by the off-the-shelf model [18].

### Method comparisons

For comparison, we benchmarked our approach against several state-of-the-art monocular depth estimation methods. The method selection is mainly guided by whether the compared methods also incorporate normal information for depth estimation with released code. Finally, the compared methods include PC-Depth [17], NASDE [14], Lite-Mono [19], and Depth Anything V2 (DAV2) [22]. Both PC-Depth and NASDE utilize surface normal information to refine depth predictions, while Lite-Mono represents a lightweight self-supervised architecture designed for efficient monocular depth estimation; DAV2 is a foundation model for monocular depth estimation. As these methods use different preprocessing strategies (e.g., image cropping, resizing), we retrained all models from scratch using our pre-processed data to ensure a consistent experimental settings. On the other hand, as all comparison methods assume a pinhole camera model, images from the C3VD dataset were pre-processed with an undistortion step to compensate for the wide field-of-view distortion present in the original frames. Specifically, OpenCV was used for image undistortion using the intrinsic and distortion parameters provided in [21]. To ensure a fair comparison, all the methods were retrained under the same settings. For DAV2, as the other methods are self-supervised and trained without ground-truth depth, we adopted the officially released pretrained model for evaluation.

**Table 1:** Quantitative comparison on SimCol3D (S) and C3VD (C) datasets

Method	Dataset	AbsRel ↓	SqRel ↓	RMSE ↓	RMSE log ↓	δ<1.25 ↑	$\delta < 1.25^2$ ↑	$\delta < 1.25^3$ ↑
PC-Depth [17]	S	0.121	0.297	1.625	0.154	0.861	0.971	0.991
DAV2 [22]		0.236	1.057	2.288	0.285	0.773	0.902	0.945
NASDE [14]		0.211	0.195	0.781	0.248	0.670	0.930	0.982
LiteMono [19]		0.098	0.076	0.443	0.129	0.931	0.980	0.991
**Ours**		**0**.**048**	**0**.**024**	**0**.**280**	**0**.**076**	**0**.**984**	**0**.**994**	**0**.**997**
PC-Depth [17]	C	0.207	1.988	8.430	0.249	0.637	0.906	0.984
DAV2 [22]		0.372	9.996	21.726	0.627	0.307	0.517	0.650
NASDE [14]		0.220	2.387	9.702	0.264	0.579	0.912	0.992
LiteMono [19]		0.185	2.826	13.168	0.234	0.660	0.939	0.991
**Ours**		**0**.**145**	**1**.**217**	**7**.**179**	**0**.**185**	**0**.**778**	**0**.**977**	**0**.**995**

**Table 2:** Quantitative comparison for generalization on C3VD dataset

Method	AbsRel ↓ ↓	SqRel ↓	RMSE ↓	RMSE log ↓	δ<1.25 ↑	$\delta < 1.25^2$ ↑	$\delta < 1.25^3$ ↑
PC-Depth [17]	0.164	1.193	6.393	0.192	0.751	0.965	0.997
NASDE [14]	0.207	2.286	9.642	0.254	0.617	0.918	0.992
LiteMono [19]	0.119	0.856	5.540	0.151	0.864	0.986	0.998
**Ours**	**0**.**110**	**0**.**708**	**5**.**181**	**0**.**138**	**0**.**896**	**0**.**991**	**0**.**999**

Experimental results on the SimCol3D and C3VD datasets are presented in Table 2, with the best results in bold.

## Results and discussion

### Comparison

*Overall performance.* Experimental results on the SimCol3D and C3VD datasets are presented in Table 1, with the best results in bold. The results demonstrate that the proposed method consistently outperformed the three comparison methods across all evaluation metrics. On the SimCol3D dataset, our approach achieved up to approximately 50% improvement on the error-based metrics compared with the state-of-the-art baselines. Particularly, DAV2 and NASDE performed worse in our experiments. This can be attributed to two main reasons: First, NASDE was originally designed for monocular depth estimation in natural scenes rather than medical endoscopy; second, NASDE relies on 3D convolutional operations, which introduce a large number of parameters and make it hard for the network to learn the mapping relations between monocular images and depths.

*Generalization ability.*

To verify the generalization ability of the proposed method, we evaluated all models trained on the SimCol3D dataset directly on the C3VD dataset without any fine-tuning. As shown in Table 2, the proposed method achieved the best performance among all comparison methods. These results demonstrate that the proposed method generalizes well across different datasets and maintains high accuracy even when tested on unseen scenarios. In addition, NASDE still performed worse, showing a similar trend to the previous comparison on overall performance. On the other hand, the models trained on SimCol3D achieved better performance on C3VD than those trained and tested on C3VD. This may be attributed to the broader range of camera motions, which provides richer supervision during training.

**Fig. 3:**
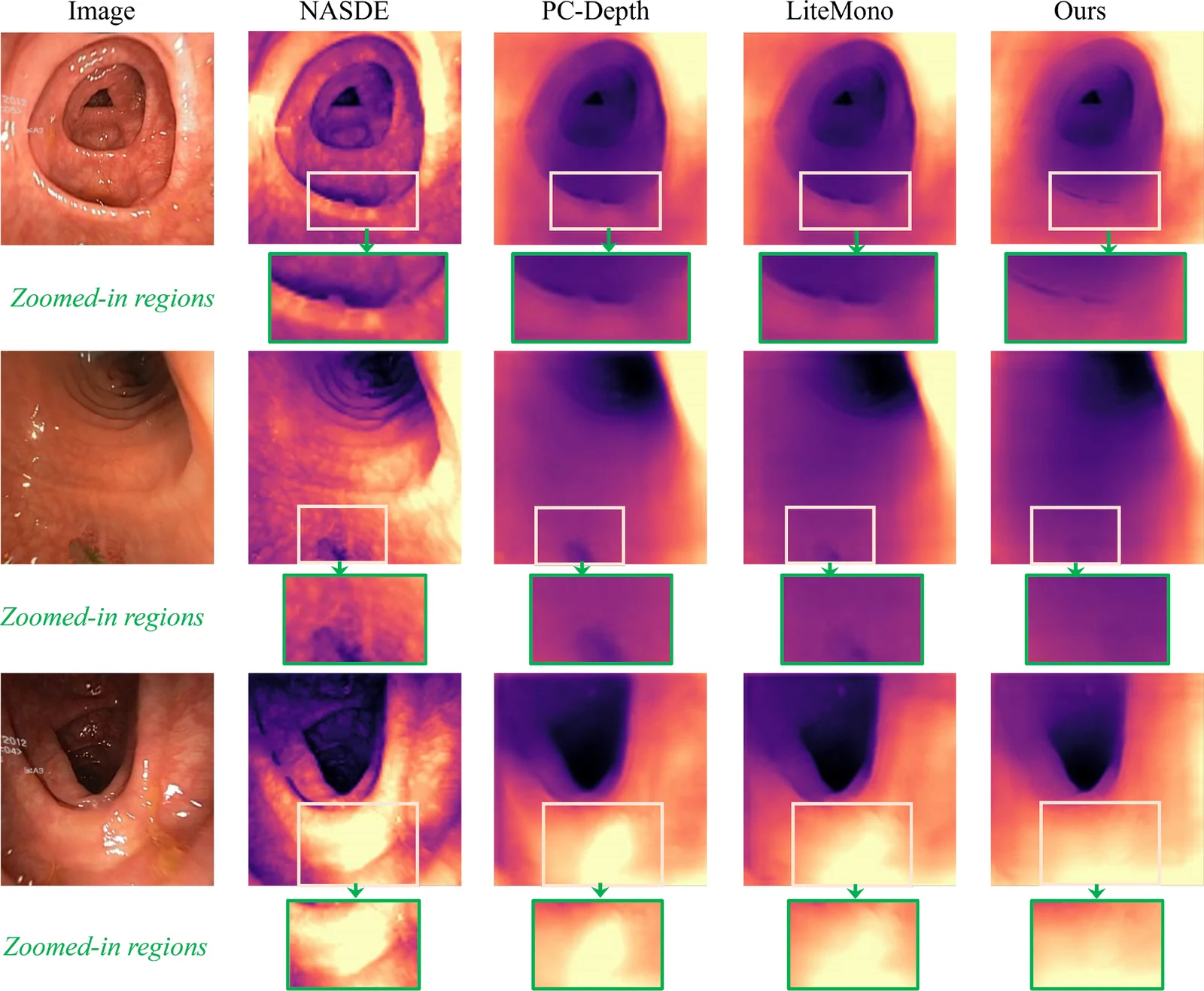
Qualitative comparison on real colonoscopy images

Furthermore, qualitative results on real colonoscopy images are presented in Fig. 3. Specifically, all methods were fine-tuned on one sequence (709 images) with 3 epochs from the HyperKvasir dataset [23] reduce the domain gap between synthetic and real data, and evaluated on separate unseen video sequences. As illustrated, the proposed method predicts more reliable depth maps than the other methods, particularly in the reflective regions of the input images. This can be attributed to the use of normal map information, which provides stronger geometric constraints and helps the model better preserve local surface structure in challenging reflective areas.

**Table 3:** Ablation studies on SimCol3D (S) and C3VD (C) datasets

Method	Dataset	AbsRel ↓	SqRel ↓	RMSE ↓	RMSE log ↓	δ<1.25 ↑	$\delta < 1.25^2$ ↑	$\delta < 1.25^3$ ↑
Baseline	S	0.098	0.076	0.443	0.129	0.931	0.980	0.991
${\text {Ours}}_{{inp}}$		0.080	0.051	0.343	0.119	0.953	0.980	0.995
${\text {Ours}}_{{feat}}$		0.073	0.044	0.336	0.100	0.963	0.989	0.995
**Ours**		**0**.**048**	**0**.**024**	**0**.**280**	**0**.**076**	**0**.**984**	**0**.**994**	**0**.**997**
Baseline	C	0.185	2.826	13.168	0.234	0.660	0.939	0.991
${\text {Ours}}_{{inp}}$		0.160	1.439	7.666	0.202	0.722	0.968	**0**.**995**
${\text {Ours}}_{{feat}}$		0.152	1.402	7.594	0.198	0.767	0.975	**0**.**995**
**Ours**		**0**.**145**	**1**.**217**	**7**.**179**	**0**.**185**	**0**.**778**	**0**.**977**	**0**.**995**

Experimental results on the SimCol3D and C3VD datasets are presented in Table 3, with the best results in bold.

### Ablation studies

To verify the effectiveness of the proposed cross-attention fusion mechanism, we compared it with two alternative methods of integrating normal and image features. The first variant, ${\text {Ours}}_{{inp}}$, performed feature fusion at the input level by directly concatenating the RGB image and the predicted surface normal maps before feeding them into the network. The second variant, ${\text {Ours}}_{{feat}}$, extracted features from the image and normal branches using separate encoders and then fuses them by element-wise addition. The variants are built on Lite-Mono due to the lightweight architecture, which served as our baseline model.

The experimental results on SimCol3D and C3VD datasets are presented in Table 3. It can be observed that: 1) all variants incorporating normal information (${\text {Ours}}_{{inp}}$ and ${\text {Ours}}_{{feat}}$) achieved better performance than the baseline, demonstrating the effectiveness of utilizing normal information to enhance depth estimation; and 2) the proposed method with the cross-attention fusion mechanism further outperformed both ${\text {Ours}}_{{inp}}$ and ${\text {Ours}}_{feat}$. This improvement may be attributed to the fact that directly concatenating normal and image inputs or performing element-wise feature addition fails to model the complex spatial correspondence and relative importance between the two modalities. In contrast, the cross-attention module enables more adaptive and geometry-aware feature interaction.

## Conclusions

In this paper, we proposed a normal-based cross-attention depth estimation model for colonoscopy. To alleviate the negative impact of specular highlights, surface normals were utilized as auxiliary geometric information, and cross-attention was employed to effectively fuse normal and image features for depth estimation. Experimental results on SimCol3D, C3VD, and real colonoscopy images from HyperKvasir dataset demonstrate that the proposed method achieves accurate depth predictions under challenging illumination and reflective conditions.
